# Calcinose tumorale d'Iclan chez un sujet âgé

**DOI:** 10.11604/pamj.2023.44.59.38797

**Published:** 2023-01-31

**Authors:** Ghita Belmaati Cherkaoui, Ayat Allah Oufkir

**Affiliations:** 1 Department of Plastic and Reconstructive Surgery, Mohamed IV Hospital University, Oujda, Morocco,; 2 Research Laboratory in Medical Sciences, Faculty of Medicine and Pharmacy of Oujda, Mohammed I university, Oujda, Morocco

**Keywords:** Calcinose, diagnostic, rare, Calcinosis, diagnosis, rare

## Abstract

Tumoural calcinosis rarely occurs in adolescents and young adults. It is characterized by a deposit of calcium in the periarticular soft tissues. We here present two characteristics and original clinical and radiographic images of sporadic left iliac cutaneous tumoural calcinosis causing non-painful swelling in an elderly woman. The study involved a 66-year old female patient with no particular past medical history who presented with polylobal erythematous mass, hard to palpation, that had evolved for 2 months (A). The diagnosis was evoked on the basis of standard frontal X-ray results (B) and biopsy results, and confirmed after histological examination of the surgical specimen. The diagnosis was made after all other possible differential diagnoses of soft tissue calcifications: calcinosis secondary to imbalances in calcium and phosphate levels, post-traumatic calcinosis and others.

## Image en médecine

La calcinose tumorale est une maladie rare de l'adolescent et de l'adulte jeune qui consiste en un dépôt de matériel calcique dans les tissus mous péri-articulaires. Nous rapportons deux images clinique et radiologique caractéristiques et originales d'une calcinose tumorale cutanée iliaque gauche sporadique, responsable d'une tuméfaction non douloureuse, chez une femme âgée. Il s'agit d'une patiente de 66 ans, sans antécédents pathologiques particuliers, consultant pour une masse érythémateuse polylobée, dure à la palpation évoluant depuis 2 mois (A). Le diagnostic est évoqué sur la radiographie standard de face (B) et sur la biopsie, et confirmé après étude histologique de la pièce d'exérèse. Le diagnostic a été porté après élimination des autres causes de calcifications des parties molles: calcinose secondaire au déséquilibre phosphocalcique, calcinose post traumatique et autres.

**Figure 1:**
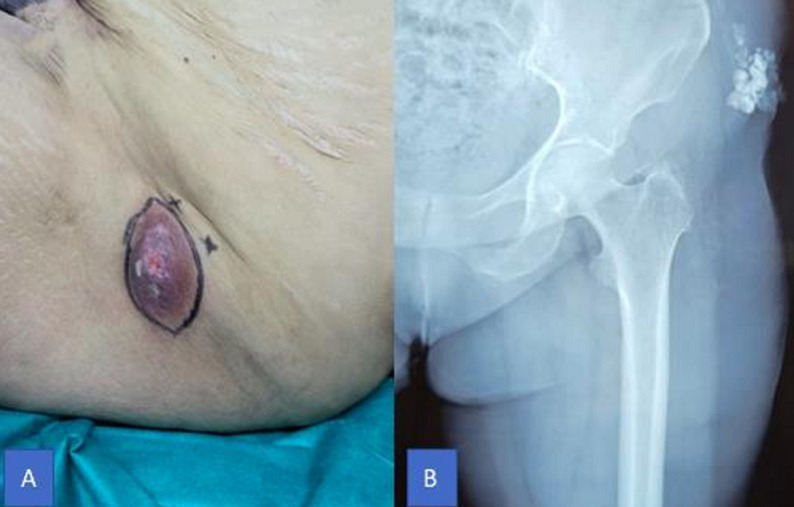
A) masse érythémateuse polylobée dure à la palpation évoluant depuis 2 mois; B) la radiographie standard de face

